# Neurotoxicities associated with checkpoint inhibitors: Two case reports and a review of the literature

**DOI:** 10.1002/ccr3.2534

**Published:** 2019-11-26

**Authors:** Lisa M. Cordes, Nicole N. Davarpanah, Lauren B. Reoma, Billel Gasmi, Martha Quezado, Omar I. Khan, Avindra Nath, Andrea B. Apolo

**Affiliations:** ^1^ Genitourinary Malignancies Branch National Cancer Institute National Institutes of Health Bethesda Maryland; ^2^ Office of Clinical Research Office of the Director National Institutes of Health Bethesda Maryland; ^3^ National Institute of Neurological Disorders and Stroke National Institutes of Health Bethesda Maryland; ^4^ Laboratory of Pathology National Cancer Institute National Institutes of Health Bethesda Maryland

**Keywords:** aseptic meningitis, checkpoint inhibitor, encephalitis, ipilimumab, neurotoxicity, nivolumab

## Abstract

We report a case of nivolumab‐induced delayed‐onset aseptic meningitis and a case of limbic encephalitis and peripheral nerve palsy with toxicity relapse 6 months after initial presentation. The atypical presentations contribute to our knowledge of these rare events and reinforce the necessity for vigilant monitoring and a multidisciplinary treatment approach.

## Graphical abstract

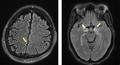

## 1 INTRODUCTION

Neurologic immune‐related adverse events are uncommon but potentially life‐threatening complications associated with immune checkpoint inhibitors. Here, we review the literature and report two cases, a rare case of nivolumab‐induced delayed‐onset aseptic meningitis and a case of limbic encephalitis and peripheral nerve palsy with toxicity relapse 6 months after initial presentation.

Within the past decade, immune checkpoint inhibitors (ICIs) have demonstrated survival advantages in various solid tumors and are now a therapeutic pillar in oncology. The primary function of immune checkpoints is to maintain immune homeostasis by down‐regulating T‐cell activation.^1^ One mechanism by which tumor cells evade the immune system is through exploiting immune checkpoints, thereby suppressing T‐cell activity.^2^, ^3^, ^4^ T‐cell anergy may be induced when programmed death‐ligand 1 (PD‐L1), primarily expressed on the tumor cell, binds to its receptor on the T cell. Blockade of this ligand‐receptor interaction may reverse immune down‐regulation, allowing for a more robust T‐cell‐mediated response. Inhibiting these negative immune regulators of T‐cell function has proven to be a successful antitumor approach.

Since 2011, 7 ICIs have been approved by the US Food and Drug Administration: ipilimumab, an inhibitor of cytotoxic T‐lymphocyte‐associated protein 4 (CTLA‐4); nivolumab, pembrolizumab, and cemiplimab‐rwlc, which inhibit programmed cell death protein 1 (PD‐1); and atezolizumab, avelumab, and durvalumab, which inhibit PD‐L1. Urothelial carcinoma (UC) is one of the many tumors that has demonstrated response to ICIs. Five PD‐1/PD‐L1 inhibitors have produced meaningful response rates in platinum‐refractory UC.^5^, ^6^, ^7^, ^8^, ^9^, ^10^ Furthermore, a significant improvement in median overall survival was demonstrated with pembrolizumab compared to chemotherapy in this population.^10^ Data also support the use of atezolizumab and pembrolizumab in chemotherapy‐naïve, cisplatin‐ineligible UC,^11^, ^12^ although the preferred first‐line treatment strategy remains controversial.^13^

Although robust data support the use of immunotherapies in many solid tumors, including UC, the efficacy of these agents in renal medullary carcinoma (RMC) is not well established. Published case reports provide a hint of activity in this rare disease.^14^, ^15^ Given the lack of standard treatment options with proven efficacy, an ICI in the setting of a clinical trial is a reasonable approach in this under‐researched population.

Severe immune‐related adverse events (irAEs), defined as grade ≥3, are estimated to occur in approximately 22%, 7.1%, and 6.3% of patients receiving a CTLA‐4, PD‐1, or PD‐L1 inhibitor, respectively.^16^ The skin, colon, endocrine organs, liver, lungs, and musculoskeletal systems are most commonly affected, although any organ system may be involved.^17^, ^18^ Despite some unique toxicities, PD‐1/PD‐L1 inhibitors have demonstrated a generally favorable toxicity profile compared to cytotoxic chemotherapy.^19^, ^20^ The incidence of any grade neurologic irAEs is estimated to be 3.8% with CTLA‐4 inhibitors, 6.1% with PD‐1 inhibitors, and 12% with the combination.^21^ Most irAEs are generally mild, with headache being predominantly reported; incidence of high‐grade events was <1%. Guillain‐Barré syndrome, myasthenia gravis, encephalopathies, and meningoradiculoneuritis are among the reported serious neurologic irAEs.^21^, ^22^ Corticosteroids remain the cornerstone of management of neurologic irAEs,^17^, ^18^, ^23^ but several cases have nevertheless proven fatal.^24^, ^25^ Select reports of autoimmune neurologic toxicities associated with ICIs are summarized in Table 1.

**Table 1:** Case Reports and Management of Select Severe Neurologic irAEs

Central Neurologic irAEs						
Neurologic irAE	Grade	Checkpoint inhibitor	Approximate time to onset	Treatment of neurologic irAE	Outcome	Reference
Meningitis						
Aseptic meningitis	2	Ipilimumab + nivolumab	1‐2 wk	No treatment, ICI held then restarted	Complete resolution	Spain et al^30^
Aseptic meningitis	3	Ipilimumab	3‐5 wk	ICI stopped, no steroids due to spontaneous symptom improvement	Complete resolution	Spain et al^30^
Aseptic meningitis	3	Ipilimumab	3‐5 wk	Stop ICI, oral prednisolone	Complete resolution	Spain et al^30^
Aseptic meningitis	N/A	Ipilimumab	4 wk	Steroids administered	Resolved	Voskens et al^31^
Aseptic meningitis	N/A	Ipilimumab (previous IL‐2)	9‐11 wk	High‐dose dexamethasone	Compete resolution	Yang et al^32^
Meningitis	N/A	Ipilimumab	1‐3 wk	Dexamethasone 8 mg/day p.o.	Complete resolution	Bot et al^24^
Meningitis	N/A	Atezolizumab	1‐3 wk	No treatment; reinitiated w/o recurrence	Symptoms resolved	Genentech^33^
Meningoencephalitis						
Meningoencephalitis	N/A	Ipilimumab + nivolumab	19 wk	ICI stopped; prednisone 100 mg/day tapered over 1 month	Full recovery	Fellner et al^35^
Meningoencephalitis	N/A	Ipilimumab + nivolumab	12 wk	ICI stopped then resumed 3 mo after symptom resolution; iv dexamethasone 10 mg twice daily for 8 d then tapered over 1 month	Full recovery	Fellner et al^35^
Herpetic meningoencephalitis	N/A	Atezolizumab	3 wk	No treatment	Patient died shortly thereafter from disease progression	Genentech^33^
Encephalitis						
Limbic encephalitis	N/A	Nivolumab	5 d	iv dexamethasone 20 mg/day tapered over 12 d then oral prednisone 10 mg/day for 14 d followed by 5 mg/day	Full recovery	Fellner et al^35^
Other						
Cerebellar ataxia and dysarthria	N/A	Pembrolizumab	29‐31 wk	ICI stopped; no treatment	Improved	Kao et al^38^
Seizure	2	Pembrolizumab (prior ipilimumab)	7 wk	Levetiracetam 500 mg twice daily	Resolved; intracerebral bleeding 3 wk later	Zimmer et al^39^
Seizure	2	Pembrolizumab	20 wk	Lorazepam	Resolved	Zimmer et al^39^
Recurring seizures; parkinsonoid/bradykinesia	2	Pembrolizumab (prior ipilimumab)	68 wk	ICI stopped; levetiracetam	Improved	Zimmer et al^39^
Meningoradiculitis	3	Nivolumab	9 wk	ICI stopped; dexamethasone 4 mg p.o. 4 times daily	Improved	Zimmer et al^39^
Cranial polyneuropathy	N/A	Ipilimumab + nivolumab	8 wk	ICI stopped; prednisone 60 mg/day then tapered over 3 mo	Full recovery	Fellner et al^35^
Phrenic nerve palsy with bulbar palsy	4	Nivolumab	7 wk	ICI stopped; methylprednisolone 1 mg/kg; IVIG; pyridostigmine	Complete resolution	Spain et al^30^

Peripheral Neurologic irAEs						
Neurologic irAE	Grade	Checkpoint inhibitor	Approximate time to onset	Treatment of neurologic irAE	Outcome	Reference
Neuromuscular Junction Disorders						
Myasthenia gravis/ paralysis (eyelids/hands)	4	Pembrolizumab (prior ipilimumab)	10 wk	Methylprednisolone 1 gram iv for 3 d; pyridostigmine 30 mg p.o. for 3 d, plasmapheresis	Not resolved; death	Zimmer et al^39^
Myasthenic crisis and polymyositis	N/A	Nivolumab	2 wk	Steroids administered; immune absorption therapy; plasma exchange therapy; IVIG	N/A	Kimura et al^40^
Demyelinating Disorders						
Polyradiculitis	N/A	Ipilimumab + nivolumab	8 wk	ICI stopped; prednisone 80 mg/day tapered over 2 mo	Full recovery	Fellner et al^35^
Polyradiculitis	N/A	Pembrolizumab	18 wk	ICI stopped; iv methylprednisolone 1 gram/day tapered over 10 d then prednisone 80 mg/day tapered over 2 mo	Partial recovery	Fellner et al^35^
Polyradiculitis	3	Pembrolizumab (prior ipilimumab)	35 wk	ICI paused; prednisolone 1 gram iv then p.o. tapering	Improved	Zimmer et al^39^
Polyneuropathy	2	Pembrolizumab	4 wk	Pregabalin 75 mg p.o. twice daily	Not resolved	Zimmer et al^39^
Polyneuropathy, worsening	2	Pembrolizumab (prior ipilimumab)	6 wk	Magnesium	Not resolved	Zimmer et al^39^
Sensorimotor neuropathy with bulbar palsy (Guillain‐Barré‐like syndrome)	4	Ipilimumab	10 wk	ICI stopped; methylprednisolone 2 mg/kg; plasmapheresis	Partial improvement	Spain et al^30^
Axonal Guillain‐Barré syndrome	5	Ipilimumab	12 wk	IVIG 0.4 gram/kg for 5 d	Death secondary to respiratory failure 3 d after starting IVIG	Bot et al^24^
Other						
Tolosa‐Hunt syndrome	N/A	Ipilimumab	18 wk	Methylprednisolone iv followed by dexamethasone p.o., local radiotherapy	Ongoing	Voskens et al^31^
Paresis, neuritis (oculomotor nerve)	2	Pembrolizumab (prior ipilimumab)	13 wk	ICI paused; prednisolone 100 mg/day	Resolved	Zimmer et al^39^
Paresis (abducens nerve, facial nerve)	3	Nivolumab (prior ipilimumab)	6 wk	ICI paused; methylprednisolone 1 mg/kg/day; IVIG	Resolved	Zimmer et al^39^
Severe necrotizing myopathy	N/A	Pembrolizumab	3‐5 wk	ICI stopped; prednisone 80 mg/day for 12 d; plasmapheresis	Death	Kao et al^38^
Facial nerve paralysis	N/A	Ipilimumab	7 wk	Steroids administered	Resolved	Voskens et al^31^

## 2 CASE PRESENTATIONS

### 2.1 Patient 1: Aseptic meningitis

A 58‐year‐old male with UC metastatic to the lung and lymph nodes who initially presented with 5 years of intermittent hematuria was found to have a left renal pelvis mass status postleft nephroureterectomy for a pT3Nx high‐grade UC followed by 4 cycles of adjuvant gemcitabine plus cisplatin with metastatic recurrence 13 months later. He was treated with 3 cycles of gemcitabine plus carboplatin without response. Relevant past medical history included obesity, noninsulin‐dependent diabetes mellitus, hypertension, hyperlipidemia, fatty liver disease, anxiety, depression, and obstructive sleep apnea. Following enrollment on a phase I clinical trial (NCT02496208), treatment was initiated with cabozantinib 40 mg p.o. once daily plus iv nivolumab 1 mg/kg every 2 weeks.

**Figure 1:**
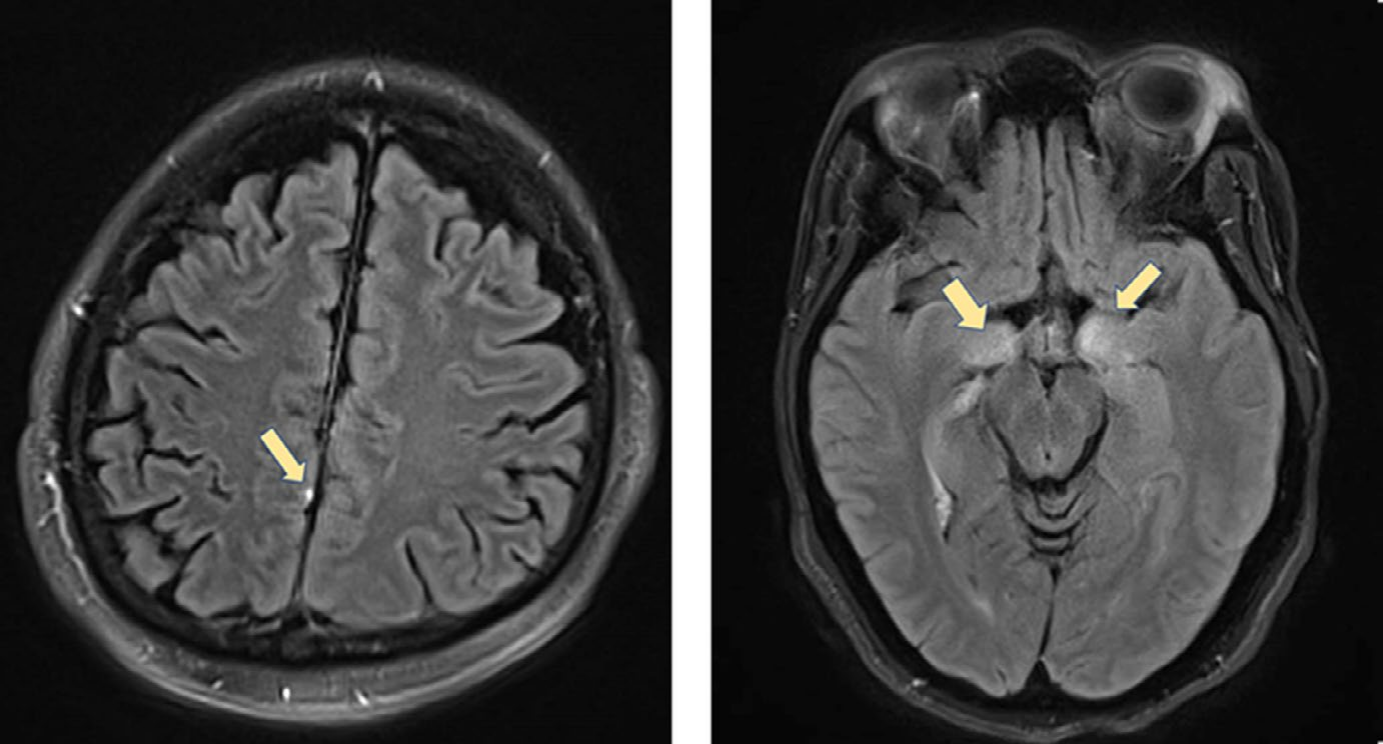
Imaging of patients 1 and 2. (Left) Meningitis, punctate focus of leptomeningeal enhancement of MRI‐Patient 1. Punctate focus of leptomeningeal enhancement on postcontrast FLAIR. (Right) Encephalitis signal abnormality in bilateral mesial temporal lobes on MRI‐Patient 2. Bilateral T2‐FLAIR hyperintense signal in the mesial temporal lobes bilaterally, with associated contrast enhancement

The first 12 cycles (48 weeks) of treatment were unremarkable. The patient achieved a complete response in the hilar and retrocrural lymph nodes and a partial response (PR) in the lung by cycle 11. Restaging evaluations at this time revealed a continuing PR to treatment, with a 51% reduction in the target right lower lobe mass and lymph node lesions. Soon thereafter, the patient presented with a fever of 39.3°C, chills, malaise, dry cough, headache, and bilateral eye pain. The patient described a dull frontal headache not relieved by analgesics and pain with ocular movement, but a neurologic examination was otherwise unremarkable. Concomitant medications were unchanged and included oxycodone, citalopram, loperamide, metoprolol succinate, opium tincture, rosuvastatin, aspirin, cholecalciferol, fexofenadine, levothyroxine, sitagliptin, and lisinopril. Treatment with nivolumab and cabozantinib was held. Three days later, the patient complained of worsening ocular pain with movement and intermittent shooting, lancinating right ear pain. MRI revealed normal orbits and a punctate focus of leptomeningeal enhancement (Figure 1). Ophthalmologic examination revealed normal vision, motility, and optic nerves. Lumbar puncture findings were consistent with aseptic meningitis (Table 2), prompting initiation of iv methylprednisolone 1 mg/kg (130 mg). Within 72 hours of starting corticosteroids, the patient experienced a rapid resolution of otic and ocular pain, fever, and headache. Methylprednisolone was transitioned to oral dexamethasone, which was then tapered over approximately 4‐5 weeks with no recurrence of symptoms. The patient was not rechallenged with nivolumab but continued cabozantinib for an additional year until disease progression in the contralateral retrocrural lymph nodes. He passed away 6 months after discontinuation of cabozantinib with extensive liver metastases. A brain autopsy showed no significant neuropathologic changes.

**Table 2:** Case Summaries of Patients 1 and 2

	Patient 1	Patient 2 Initial presentation	Patient 2 Relapse
Immunotherapy	Nivolumab × 24 doses	Ipilimumab/nivolumab x 4 doses then nivolumab x 4 doses	None since initial treatment
Neurologic autoimmune diagnosis	Aseptic meningitis (grade 3)	Limbic encephalitis (grade 3)	Relapsed limbic encephalitis (grade 3)
Presenting symptoms	Fever, chills, malaise, dry cough, headache, bilateral eye pain, and right ear pain	Blurry vision, headache, photophobia, and short‐term memory impairment	Confusion, paranoia, and short‐term memory impairment
Neurologic examination findings	Unremarkable	Anterior bilateral uveitis (2 mo prior) (grade 1), ataxia, Impaired delayed recall, right 6th cranial nerve palsy	Left 6th cranial nerve palsy, bilateral ptosis^a^, MoCA = 11/30
Brain MRI results	Punctate focus of leptomeningeal enhancement	FLAIR hyperintensities in bilateral mesial temporal lobes	Bilateral lateral rectus atrophy
Lumbar puncture results	Protein 64 mg/dL Glucose 56 mg/dL RBC 1/mm^3^ WBC 74/mm^3^ (91% lymphocytes) Opening pressure 21 mm H_2_O No organisms/growth Cytopathology negative	Protein 34 mg/dL Glucose 53 mg/DL RBC 0/mm3 WBC 19/mm3 (99% lymphocytes) No organisms/growth Cytopathology negative Paraneoplastic panel negative AChR negative MuSK negative	Protein 57 mg/dL Glucose 48 mg/dL RBC 0/mm^3^ WBC 4/mm^3^ NMDA receptor antibody negative bParaneoplastic, autoantibody panel negative Cytopathology negative No organisms/growth Pattern 4 oligoclonal bands
Additional findings/ assessments			EEG showed intermittent focal delta slowing in the bilateral frontal region
irAE treatment	Methylprednisolone 1 mg/kg iv x 1 then dexamethasone p.o. taper over 4‐5 wk	Methylprednisolone 1 gram iv daily x 5 then prednisone p.o. taper over 3 mo; mycophenolate unsuccessful	Methylprednisolone 1 gram iv daily x 5 then prednisone oral taper over 4 mo
irAE outcome	No sequelae	Partial improvement	Partial improvement

Abbreviations: AChR, antiacetylcholine receptor antibody; EEG, electroencephalogram; FLAIR, fluid‐attenuated inversion recovery; iv, intravenous; irAE, immune‐related adverse event; LP, lumbar puncture; MoCA, Montreal Cognitive Assessment; MuSK, muscle‐specific receptor kinase; NMDA, N‐methyl‐D‐aspartate; RBC, red blood cell; and WBC, white blood cell.

a Initially presented with left ptosis and progressed to bilateral ptosis.

b VGKC‐complex Ab IPA, LGI1‐IgG CBA, CASPR2‐IgG CBA, GAD65 Ab Assay, GABA‐B‐R Ab CBA, AMPA‐R Ab CBA, ANNA‐1‐3, AGNA‐1, PCA‐1 and 2, PCA‐Tr, Amphiphysin Ab, CRMP‐5‐IgG

### 2.2 Patient 2: Limbic encephalitis

A 42‐year‐old female with newly diagnosed metastatic RMC to the bilateral lungs, bulky retroperitoneal lymph nodes, and multiple subcutaneous nodules presented for consultation and treatment initiation. Relevant past medical history included sickle cell trait; concomitant medications included oxycodone, acetaminophen, docusate/senna, and ondansetron. Following consent, treatment with immunotherapy and a targeted therapy were initiated based on the results of a published, ongoing clinical trial.^26^ Treatment consisted of cabozantinib 40 mg p.o. once daily and iv nivolumab 3 mg/kg plus iv ipilimumab 1 mg/kg every 3 weeks for 4 cycles, followed by cabozantinib 40 mg p.o. once daily and iv nivolumab 3 mg/kg every 2 weeks as maintenance therapy.

The patient experienced a PR following 7 cycles (approximately 4 months) of therapy. A biopsy of a residual subcutaneous nodule revealed necrotic tissue. Treatment was complicated with bilateral anterior uveitis that presented as blurry vision and resolved with topical agents. A short time later, the patient presented with a 5‐day history of diplopia, headache, photophobia, and difficulty with short‐term memory. Neurologic examination was significant for impaired delayed recall, right 6th cranial nerve palsy, and ataxia. MRI revealed a signal abnormality in bilateral mesial temporal lobes (Figure 1). Findings of a lumbar puncture and additional tests are included in Table 2. The patient experienced partial symptom improvement with methylprednisolone 1 gram iv daily for 5 days, followed by a prednisone taper. Approximately 2 weeks later, the patient developed acute bilateral intermittent ptosis during a prednisone dose reduction from 80 mg to 40 mg daily. Prednisone was re‐escalated, followed by a slower taper over approximately 3 months. A trial of mycophenolate also occurred during the taper period but without apparent symptomatic benefit.

**Figure 2:**
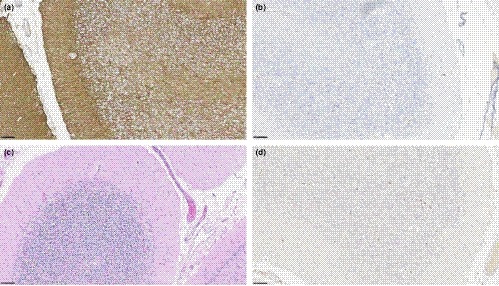
Pathologic findings in cerebellum on autopsy for patient 2. A, GFAP 20×, B, CD68 20×, C, HE 20×, and D, CD3 20×

The patient completed the steroid taper and was not rechallenged with immunotherapy. She remained neurologically stable until approximately 6 months after the initial presentation, at which time she presented with a 2‐week history of confusion, paranoia, and short‐term memory impairment. Neurologic examination revealed symptoms of contralateral left 6th cranial nerve palsy and a Montreal Cognitive Assessment of 11/30. MRI was notable for progression of atrophy but no alteration or enhancement to account for the new‐onset palsy. Cerebrospinal fluid (CSF) analysis is provided in Table 2. The recurrent symptom exacerbation was attributed to a relapse of autoimmune encephalitis, and the patient was treated with another round of methylprednisolone 1 gram iv daily for 5 days, followed by a slow (4‐month) prednisone taper. Similar to the first occurrence, the patient experienced a partial resolution of symptoms, but signs of neurocognitive slowing remained. The patient died 10 months later. At autopsy, gross examination of the brain showed no macroscopic abnormalities. Selected regions of the brain were examined with hematoxylin and eosin and various immunohistochemical stains (GFAP, NEUN, LFB‐PAS, NFTP, CD68, CD3, CD4, and CD8; Figure 2). Sections of the cerebellum show a marked loss of Purkinje cells, accompanied by microglial activation and Bergman gliosis. Sparse inflammatory cells mainly consist of scattered lymphocytes and macrophages. Additional changes of microglial activation and gliosis are seen in other regions, including the hippocampus.

## 3 DISCUSSIONS AND CONCLUSIONS

We present 2 cases of serious neurologic irAEs, aseptic meningitis and limbic encephalitis, both associated with the use of ICIs. Both patients had remarkable durable disease responses to immunotherapy. The patient with UC experienced a durable (approximately 2 years) PR on the nivolumab/cabozantinib regimen. Similarly, the patient with RMC experienced a PR on the nivolumab/ipilimumab/cabozantinib combination for approximately 5 months. Emerging data have begun to establish a correlation between irAEs and the efficacy of ICIs in patients with solid tumors^27^, ^28^; our cases provide additional evidence for this correlation.

Drug‐induced aseptic meningitis is a rare adverse event that has been reported with various pharmaceutical products, including nonsteroidal anti‐inflammatory drugs, antimicrobials, iv immunoglobulin (IVIG), vaccines,^29^ and most recently, ICIs.^24^, ^30^, ^31^, ^32^, ^33^ Patient 1 denied use of these agents, and there are no data supporting a link between cabozantinib and aseptic meningitis. Furthermore, viral, bacterial, and fungal causes of meningitis were ruled out. Presenting symptoms of fever and headache are classic signs of aseptic meningitis, but our patient also complained of ocular and otic pain. The reported median time to onset of neurologic irAEs is 6 weeks.^21^ Onset of meningitis‐associated symptoms in our patient was much later compared to previously published reports (24 doses vs 1‐2 doses, respectively).^24^, ^30^, ^31^, ^32^, ^33^ Drug‐induced aseptic meningitis may be self‐limited and resolve without treatment. Case reports of aseptic meningitis associated with ICIs have also demonstrated spontaneous symptom resolution.^30^, ^33^ Current guidelines recommend monitoring of steroids or considering steroids for moderate/severe symptoms or unwell patients.^17^, ^23^ Due to progressive symptoms during an initial work‐up period, we elected to initiate steroids in our patient.

Patient 2 met the diagnostic criteria for autoimmune limbic encephalitis^34^: subacute onset of memory deficits; fluid‐attenuated inversion recovery hyperintensities in bilateral medial temporal lobes; CSF pleocytosis; and a reasonable exclusion of alternative causes. One case of limbic encephalitis associated with nivolumab reported a symptom onset of 5 days followed by a full recovery with steroid treatment.^35^ To our knowledge, this is the first case of ICI‐associated limbic encephalitis with recurrent symptoms months after initial presentation. However, the patient did not fully recover to baseline cognition after the first neurologic irAE. Theoretically, the symptoms may have been suppressed by high‐dose steroids and a 3‐month taper and later progressed after steroid discontinuation. High‐level evidence for the treatment of immunotherapy‐associated encephalitis is lacking, and practice recommendations are based on case reports and/or expert opinion. Current guidelines recommend a trial of methylprednisolone 1‐2 mg/kg,^17^, ^18^, ^23^ and suggest methylprednisolone 1 gram iv daily for 3‐5 days plus IVIG for patients with severe or progressing symptoms or with oligoclonal bands.^23^ A further treatment option is plasmapheresis.^23^ Our patient was treated with pulse methylprednisolone 1 gram iv daily for 5 days for both the initial presentation and the recurrent episode, which improved but did not eradicate her cognitive symptoms. A trial with mycophenolate, a corticosteroid‐sparing agent, was unsuccessful.

On brain autopsy, sections of patient 2's cerebellum showed a marked loss of Purkinje cells accompanied by microglial activation and Bergman gliosis. The cerebellum is a frequent target of paraneoplastic autoimmunity. With paraneoplastic cerebellar degeneration (PCD), pathologic examination shows marked degeneration of Purkinje cells with minimal involvement of the molecular or granular cell layers. Depending on disease stage, inflammation can be marked, sparse, or absent in the cerebellar cortex.^36^ Inflammation in other regions of the brain can also be seen and might indicate an overlap with paraneoplastic encephalomyelitis. Three antibodies, anti‐Yo, anti‐Tr, and anti‐mGluR1, are predominantly associated with cerebellar dysfunction. In addition to antibody‐mediated immune responses, cytotoxic T‐cell responses are involved in the pathogenesis of PCD.^37^ In this case, anti‐Purkinje antibody titers performed a year prior to death were negative, but in the absence of concurrent antibody titers, we cannot rule out the possibility of a paraneoplastic syndrome. Also, given the patient's prior treatment with ICIs, the possibility of irAEs should be considered.

Although reports of aseptic meningitis and limbic encephalitis after treatment with ICIs have previously been published, these cases help expand our knowledge of these rare but potentially serious neurologic toxicities. Patient 1 provides evidence of an unusual late‐onset aseptic meningitis following 24 doses of nivolumab. Patient 2 is a unique case of recurrent limbic encephalitis. Neurologic sequelae from these agents may occur, requiring prompt attention and a multidisciplinary approach to reduce morbidity and mortality.

## CONFLICT OF INTEREST

None declared.

## AUTHOR CONTRIBUTIONS

LMC, NND, and ABA: made major contributions to the writing of this manuscript. All authors read and approved the final manuscript.
